# Fatal cardiac tamponade due to coronary sinus thrombosis in acute lymphoblastic leukaemia: a case report

**DOI:** 10.1186/1757-1626-2-9095

**Published:** 2009-11-27

**Authors:** Sohei Kitazawa, Riko Kitazawa, Takeshi Kondo, Kiyoshi Mori, Toshimitsu Matsui, Hiroshi Watanabe, Makoto Watanabe

**Affiliations:** 1 Division of Diagnostic Molecular Pathology, Kobe University Graduate School of Medicine, 7-5-1 Kusunoki-cho, Chuo-ku, Kobe 650-0017, Japan; 2 Division of Haematology, Kobe University Graduate School of Medicine, 7-5-1 Kusunoki-cho, Chuo-ku, Kobe 650-0017, Japan; 3 Faculty of Health Sciences, Kobe University School of Medicine, 7-10-2 Tomogaoka, Suma-ku, Kobe 654-0142, Japan

## Abstract

We report a rare case of fatal cardiac tamponade attributed to coronary sinus thrombosis. An 83-year-old man was admitted to the hospital complaining of general fatigue. Laboratory examination revealed marked increase of atypical lymphoblastic cells in peripheral blood. CHOP therapy was started under the diagnosis of acute lymphoblastic leukemia. The patient died, however, of sudden cardiac arrest in the initial course of the chemotherapy. Autopsy revealed cardiac tamponade with markedly dilated and congested coronary vein induced by coronary sinus thrombosis. A condition similar to leukemia-related venous thromboembolic disease, combined with endothelial damage induced by leukemic infiltration, may cause this rare complication.

## Introduction

Coronary sinus thrombosis (CST) is an unusual but serious complication of central venous catheter devices [1]. Direct trauma of the catheter to the coronary sinus endothelium is the most common cause of the thrombosis [1,2]. The clinical outcome of CST is often unpredictable, however, and sometimes even asymptomatic [3] due to the rapid recovery of blood flow by collateral circulation. Although not so common as rupture of the aneurysm of sinus of Valsalva, CST can cause pericardial tamponade leading to sudden death [4,5]. Here, a rare case of fatal cardiac tamponade attributed to coronary sinus thrombosis in an 83-year-old man with acute lymphoblastic leukemia is described.

## Case presentation

**Figure 1:**
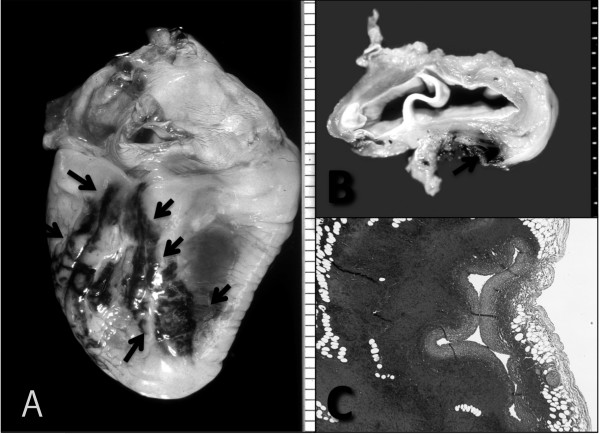
**Macroscopic and microscopic findings of the heart**. (A) The heart weighted 460 g and showed marked dilated and congested coronary veins posterior to the right ventricle (arrows). (B). A formalin-fixed horizontal cross-section of the base of the heart revealed a fresh thrombus at the orifice of the coronary sinus (arrow). (C) Histopathological examination revealed extensive hemorrhagic change around the coronary vein (HE, ×40). At the site of venous rupture, infiltration of leukemic cells was focally observed.

**Figure 2:**
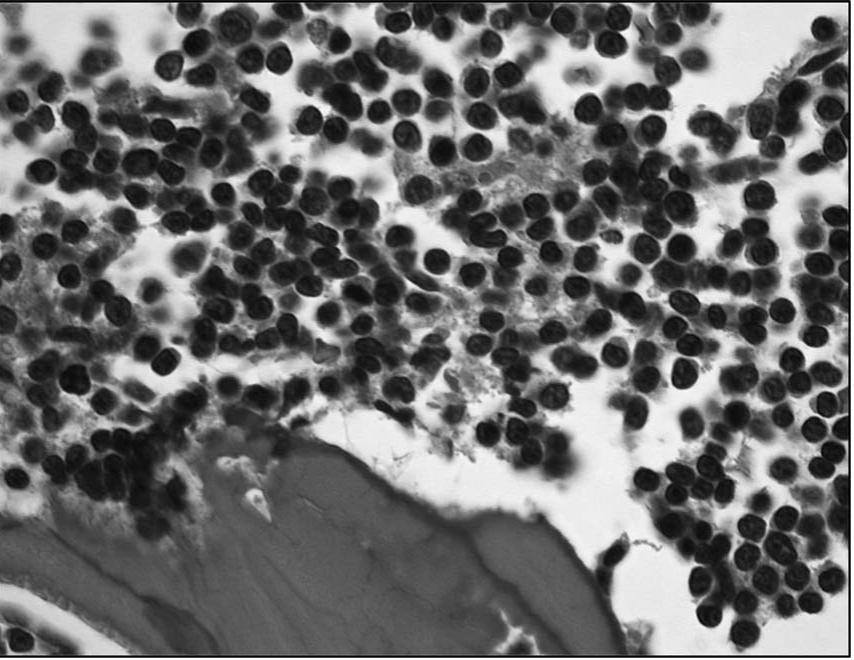
**Lymphoblastic leukaemia infiltrating the bone marrow cavity**. Leukemic cells, with small round nuclei, diffuse and dense chromatin content and scant cytoplasm, are seen infiltrating the bone marrow cavity (HE, ×400).

An 83-year-old Japanese man was admitted to the hospital complaining of general fatigue. Laboratory examination revealed marked increase of atypical lymphoblastic cells in peripheral blood. The diagnosis of acute lymphoblastic leukaemia was made, and combined chemotherapy (CHOP therapy) was started. During the initial course of chemotherapy, however, the patient suffered sudden cardiac arrest and, despite undergoing intensive efforts at resuscitation, died soon after the onset of symptoms; the cause of cardiac arrest could not be ascertained. At autopsy twelve hours after death, accumulation of 400 ml of fresh blood fluid was noted in the pericardiac space. The heart, weighing 460 g, showed markedly dilated and congested coronary veins (Figure 1A arrows). A horizontal cross-section of the base of the heart revealed a fresh thrombus at the orifice of the coronary sinus (Figure 1B, arrow). Histologic examination revealed extensive hemorrhagic change around the coronary vein (Figure 1C, HE, ×40). Infiltration of leukemic cells was focally observed at the site of venous rupture; the cells, with small round nuclei, diffuse and dense chromatin content and scant cytoplasm, infiltrated almost all the organs, including the bone marrow cavity (Figure 2, HE, ×400). The final diagnosis of cardiac tamponade attributed to coronary sinus thrombosis was established histopathologically.

## Discussion

Except for a very rare and spontaneous primary case [6], CST is usually initiated by endothelial damage after access to the right atrium through invasive cardiac procedures such as insertion of central venous lines, pacing wire, or coronary sinus catheterization [1,2]. It has also been documented as a complication of heart transplants, mitral valve replacement and infectious endocarditis [3]. Similar to venous thromboses, other than vessel wall injury leading to endothelial damage, factors such as stasis and alteration of the coagulation status all contribute to the formation of CST.

Thromboembolic complications in malignancies include clinically silent hemostatic abnormalities, venous thromboembolism, pulmonary embolism, disseminated intravascular coagulation and life-threatening thrombohemorrhagic syndrome [7]. While cerebral venous and sinus thromboses are well documented as relatively rare but often fatal forms of venous thromboembolic complications of hematological malignancies [8], CST with sudden cardiac arrest after acute pericardial tamponade in leukaemic patients has not been reported. Because both endothelial damage by leukaemic cell infiltration to the venous vessel wall and the hyperviscosity and hypercoagulation status by leukaemic cells are common in leukaemia, especially in acute lymphoblastic leukaemia, clinically silent CST or sudden death by CST, as seen in this case, may be either missed or clinically not recognized as such. Furthermore, this sudden thrombotic risk could be increased by antiblastic drugs affecting the procoagulant activity of cells and the production of coagulation inhibitors from the liver [9].

## Consent

Written informed consent was obtained from the patient for publication of this case report with accompanying images. A copy of the written consent is available for viewing by the Editor-in-Chief of the journal.

## Competing interests

The authors declare that they have no competing interests.

## Authors' contributions

All authors analyzed and interpreted the patient data regarding the hematological disease and the autopsy. SK and RK conducted the histological examinations and were major contributors in writing the manuscript. All authors read and approved the final manuscript
